# Supplementary material for: Astroglial exosome HepaCAM signaling and ApoE antagonization coordinates early postnatal cortical pyramidal neuronal axon growth and dendritic spine formation
Source: Nat Commun. 2023 Aug 24;14:5150. doi: 10.1038/s41467-023-40926-2 (PMC10449881; doi:10.1038/s41467-023-40926-2)
Supplement: Supplementary file 1 — Supplementary information [file 41467_2023_40926_MOESM1_ESM.pdf]

Supplementary Information

Astroglial exosome HepaCAM signaling and ApoE antagonization coordinates early postnatal cortical pyramidal neuronal axon growth and dendritic spine formation

Jin S et al.,

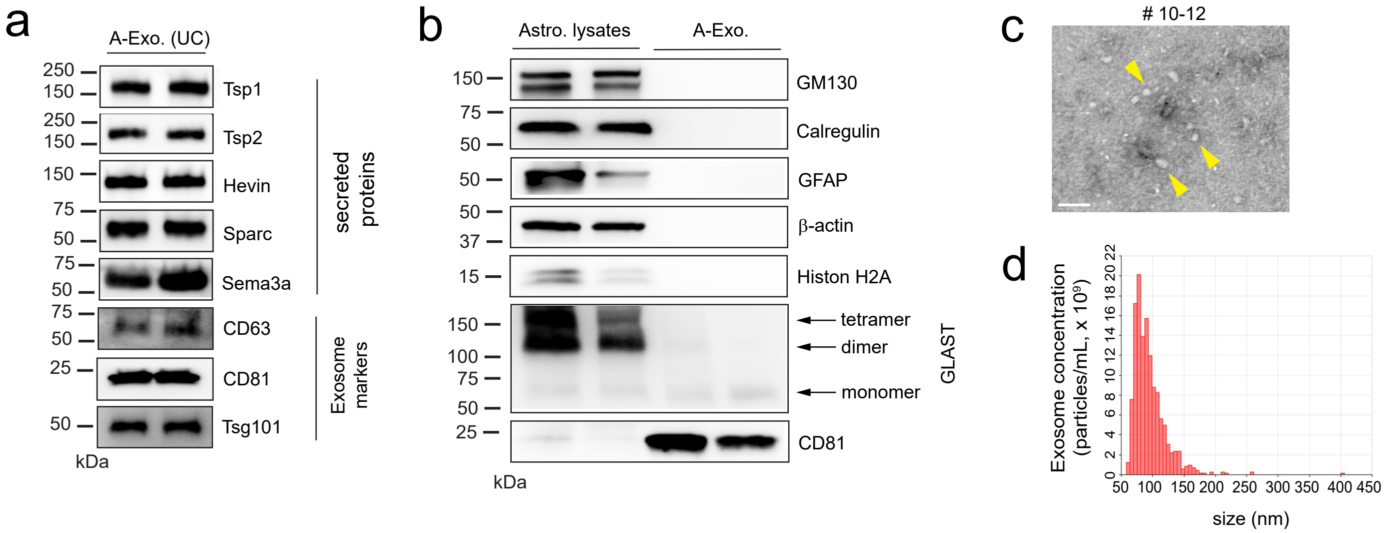

Supplementary Figure 1

Supplementary Fig. 1 Selective isolation of astroglial exosomes (A-Exo.) from ACM using the size exclusion chromatography (SEC)

a, Representative (from 3 replicates) immunoblots of astroglia secreted proteins and exosome markers in A-Exo. isolated from ACM (20 mL/sample) by ultracentrifυγation (UC). **b**, Representative (from 3 replicates) immunoblots of typical protein markers for exosomes (CD81), other subcellular organelles (GM130 for Golgi apparatus; Calregulin for ER; Histon H2A for nucleus), and cytoskeleton (GFAP/β-actin) and plasma membrane proteins (GLAST) from astrocytic lysates and purified exosome fractions of ACM. **c,** Representative (from 3 replicates) immunoEM images of CD63 labeling in SEC eluted fractions #10-12; yellow arrows: $CD63^-$ small vesicles; scale bar: 100 nm. **d**, Representative size distribution analysis of A-Exo. measured by the qNano particle analyzer.

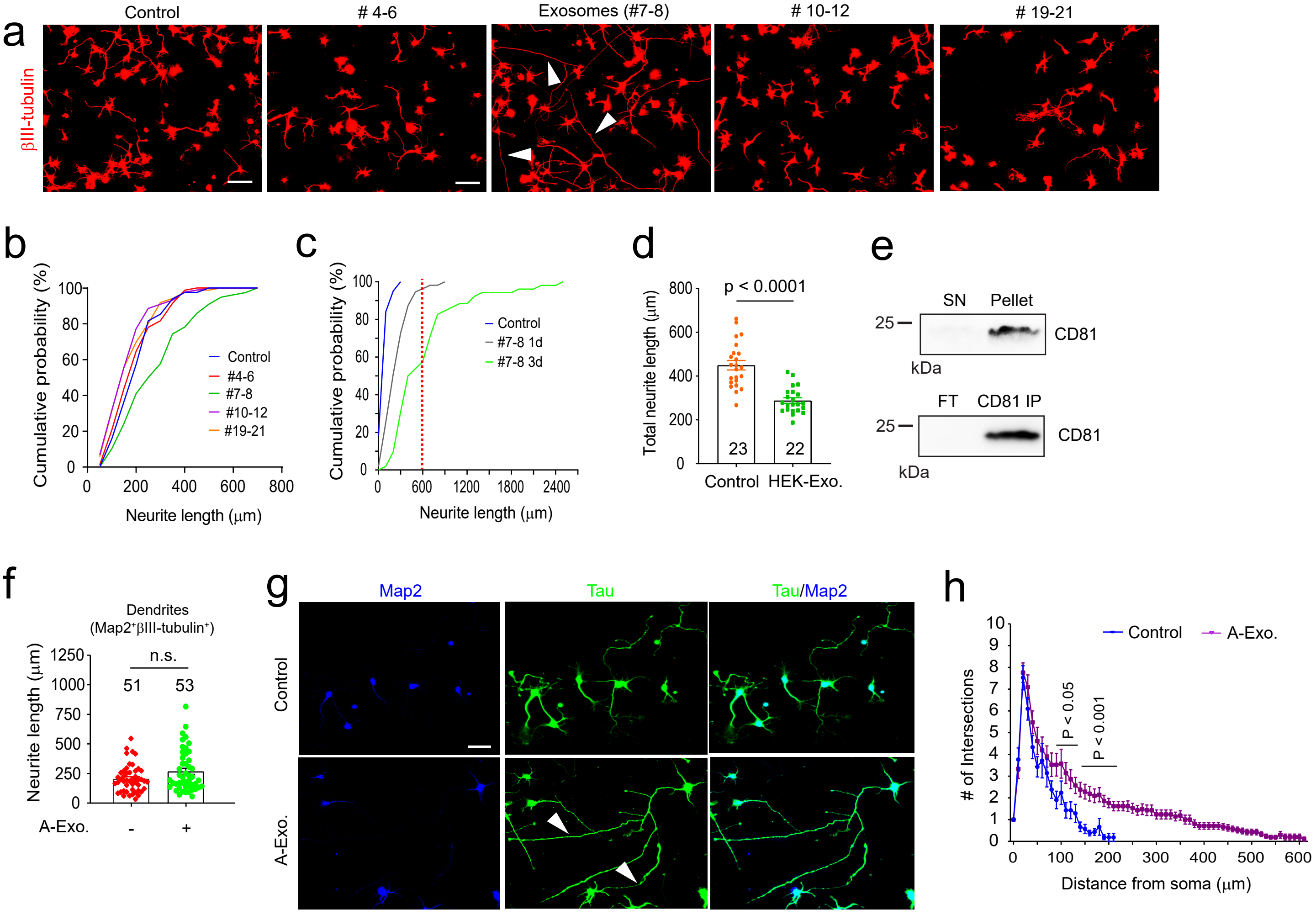

Supplementary Figure 2

Supplementary Fig. 2 Size exclusion chromatography (SEC)-isolated astroglial exosomes (A-Exo.) selectively stimulate neuronal axon growth

a, Representative images of βIII-tubulin^{+} cortical neurons following treatment with eluted fractions (pooled as indicated) #4-6, #7-8, #10-12, or #19-21 from the SEC. Scale bar: 100 μm; **b**, Quantification of total neurite length of cortical neurons following treatment with eluted fractions (pooled as indicated, 100 μl). #4-6 (no protein detected), #10-12, and #19-21 (1μg/μl) from the SEC of ACM (initial 100 mL). 1μg exosomes (#7-8) were used in treatment, n = 78-88 neurons (10-12 neurons/replicate, > 3 biological replicates)/group; **c**, Quantification of total neurite length of cortical neurons following treatment with fractions #7-8 (5μl, 0.2 μg/μl) for 1 or 3 d. n = 52-82 neurons (10-12 neurons/replicate, > 3 biological replicates)/group; **d**, Quantification of total neurite length of cortical neurons following treatment with HEK exosomes isolated by SEC. Number of neurons quantified in each group shown in the graph (6-8 neurons/replicate, 3 biological replicates)/group; **e**, Representative (from 3 replicates) immunoblot of CD81 in the supernatant (SN) or pellet of SEC fractions #7-8 (1 mL, from initial 10 mL ACM) following an additional 24 h ultracentrifugation (UC, 100,000 x g), or in the flowthrough (FT) or CD81 immunoprecipitation (IP) pellet of SEC fractions #7-8 after CD81 pull-down. **f**, Quantification of dendrite (Map2^{+}βIII-tubulin^{+}) length of cortical neurons following A-Exo. treatment. Number of neurons quantified in each group shown in the graph (10-12 neurons/replicate, > 3 biological replicates)/group; **g**, Representative images of Map2 and Tau staining on cortical neurons following A-Exo treatment. Scale bar: 50 μm; **h**, Sholl analysis of cortical neurons following A-Exo treatment. n = 21 neurons (7 neurons/replicate, 3 biological replicates)/group; 1 μg exosome was used

in **b-c, f,** and **h**. p values in **d** and **f** determined from two-tailed t test. p value in **h** determined by multiple unpaired two-tailed t test. Data are presented as mean values +/- SEM.

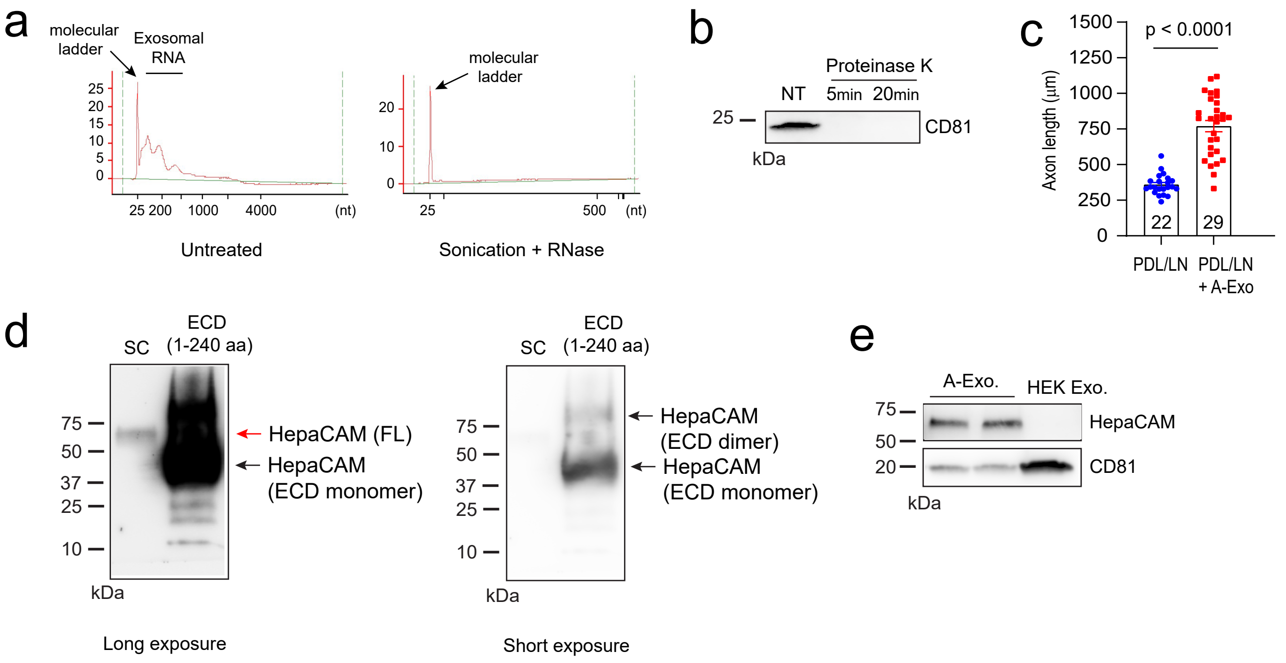

Supplementary Figure 3

Supplementary Fig. 3 Surface signaling is essential and sufficient to mediate stimulatory effects of A-Exo. on axon growth

a, Representative (from 3 replicates) bioanalyzer tracer of exosomal RNA with and without RNase treatment (5 minutes) following sonication. Sufficient small RNA was observed in untreated A-Exo. **b**, Representative (from 2 replicates) immunoblot of CD81 following proteinase K treatment. 0.5 μg A-Exo. was treated with proteinase K for either 5 or 20 minutes. NT: not treated A-Exo; CD81 immunoreactivity disappeared from the immunoblot as a result of the proteinase K digestion; **c**, Quantification of axon length of cortical neurons plated on either PDL/laminin (LN) coated or PDL/LN/A-Exo. coated coverslips. Number of neurons quantified in each group shown in the graph (7-10 neurons/replicate, ≥ 2 biological replicates)/group; 1μg A-Exo. was used in each treatment. p value in **c** determined from two-tailed t test; Data are presented as mean values +/- SEM. **d**, Representative (from 3 replicates) HepaCAM immunoblot with spinal cord (sc) lysate (20 μg) and recombinant human HepaCAM extracellular domain (ECD) protein (1-240 aa, 1 μg). HepaCAM antibody (Proteintech) is able to detect mouse HepaCAM full-length (sc lane) and human ECD (monomer and dimer). **e**, Representative (from 3 replicates) HepaCAM immunoblot in A-Exo. and HEK exosomes.

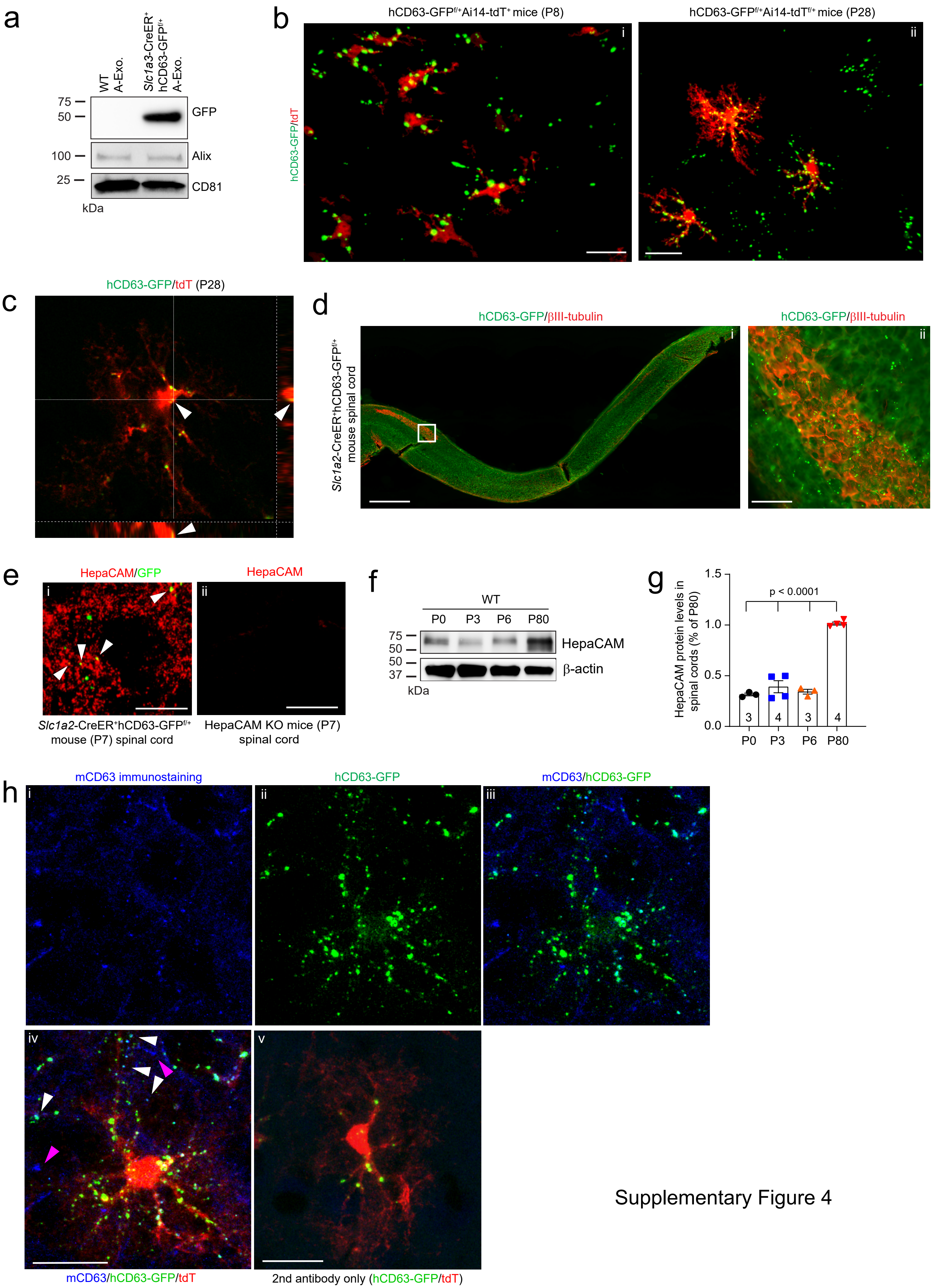

Supplementary Figure 4

Supplementary Fig. 4 a, ***In situ* illustration of A-Exo. in the CNS**

a, Representative (from 5 replicates) immunoblots of GFP tag and exosome markers Alix and CD81 on A-Exo. collected from WT and *Slc1a3*-$CreER^{+}$hCD63-$GFP^{f/+}$ astrocyte cultures; 4-OHT (final conc.: 1μM) was added onto *Slc1a3*-$CreER^{+}$hCD63-$GFP^{f/+}$ astrocyte cultures at DIV 3; **b**, Representative (from > 5 injected mice) images of tdT^{+} astroglia and astroglia-derived hCD63-GFP^{+} puncta from the motor cortex of AAV5-mCherry-*Gfap*-Cre-injected hCD63-$GFP^{f/+}$Ai14-$tdT^{f/+}$ mice at P8 (i) and P28 (ii). Scale bar: 20 μm; **c**, Representative Orthogonal view of hCD63-GFP signal inside tdT^{+} astroglia from the P28 image in Fig. 4b iii; **d**, Representative (from > 4 mice) longitudinal image of βIII-tubulin staining and astroglia-derived hCD63-GFP^{+} puncta along the spinal cord from 4-OHT-injected *Slc1a3*-$CreER^{+}$ mice at P8. Subpanel i: the longitudinal image of the spinal cord; Subpanel ii: a magnified view of the box in the subpanel i; Scale bar: 1mm (subpanel i); 100 μm (subpanel ii); **e**, Representative image of HepaCAM immunostaining signals co-localized (white arrows) with hCD63-GFP^{+} puncta signals from spinal cord sections of 4-OHT-injected *Slc1a3*-$CreER^{+}$hCD63-$GFP^{f/+}$ mice (P7, subpanel i). Scale bar: 50μm; No HepaCAM immunoreactivity signals were detected in hepaCAM KO spinal cords (subpanel ii). Representative HepaCAM immunoblot (**f**) and quantification (**g**) of HepaCAM expression in spinal cords during postnatal development. n = 3 mice for P0 and P6, 4 mice for P3 and P80; p values determined by one-way ANOVA followed by post-hoc Tukey's test. Data are presented as mean values +/- SEM. **h**, Immunostaining (from 2 replicates) of endogenous mouse CD63 on the cortex of AAV5-mCherry-*Gfap*-Cre-injected hCD63-$GFP^{f/+}$Ai14-$tdT^{f/+}$ mice. Subpanels i: mouse (m) CD63-GFP fluorescence; ii: hCD63-GFP fluorescence; iii, merge of hCD63-GFP fluorescence and mCD63 immunostaining; iv: merge of hCD63-GFP, tdT fluorescence, and mCD63

immunostaining; white arrows: co-localized hCD63-GFP fluorescence and mCD63 immunoreactivity; purple arrows: mCD63 immunoreactivity only; v: 2nd antibody only with tdT and hCD63-GFP fluorescence; Scale bars in iv and v: 20μm.

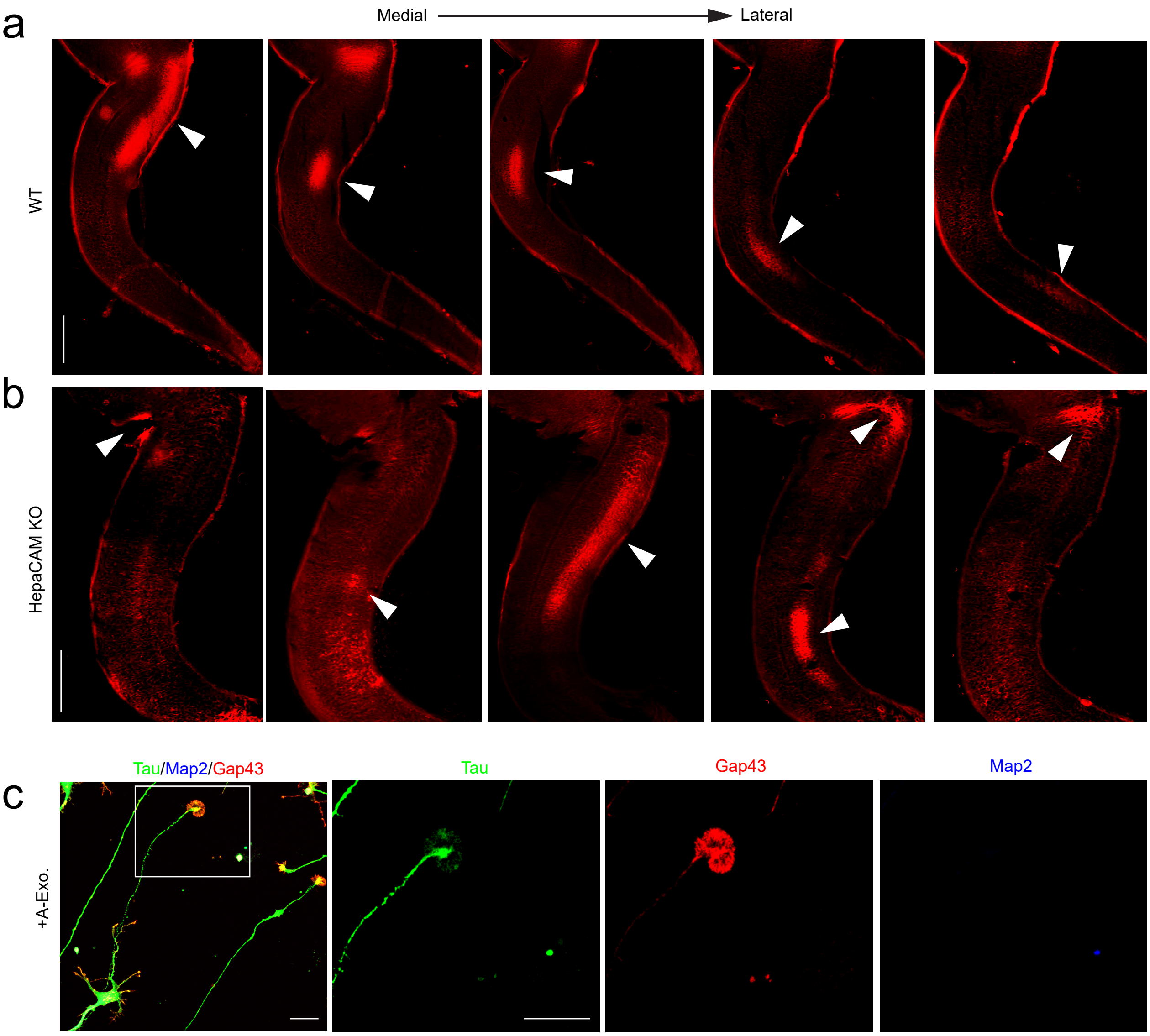

Supplementary Figure 5

Supplementary Fig. 5 Labeling of corticospinal tract (CST) on longitudinal sections of the spinal cord following CM-DiI injections

Representative original set of longitudinal images from CM-DiI-injected WT (**a**) and HepaCAM KO (**b**) mouse spinal cords that were superimposed into the continuous CST axon growth image shown in Fig. 5C. Images of longitudinal spinal cord sections were taken from lateral to medial orientation at P3. White arrows: CM-DiI labeling; Scale bar: 1mm; **c**, Representative (from > 10 replicates) image of Tau, Map2, and Gap43 immunostaining of A-Exo-treated cultured cortical neurons to illustrate axonal growth cones and axons; Scale bar: 20 μm.

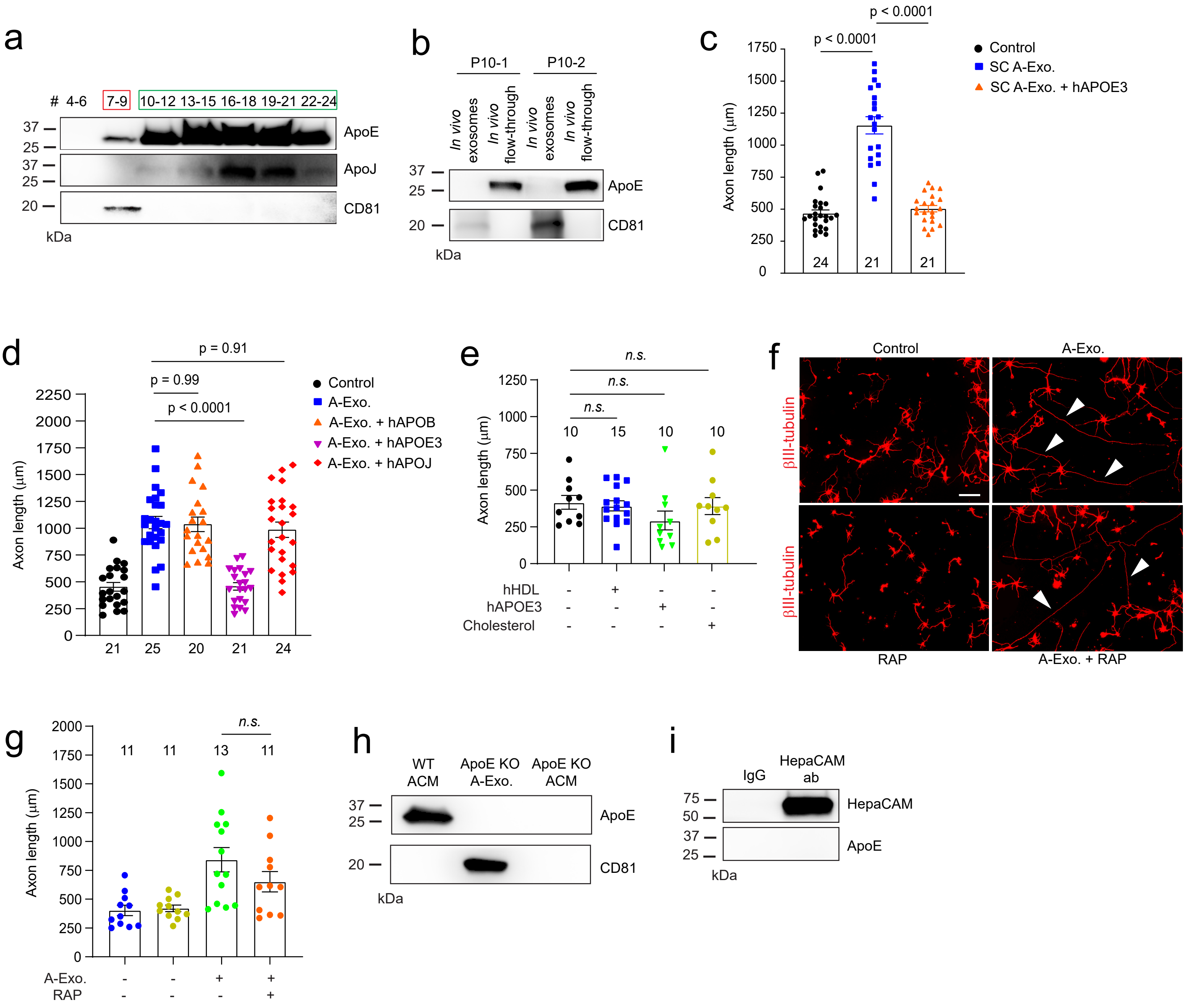

Supplementary Figure 6

Supplementary Fig. 6 ApoE/cholesterol is not involved in the stimulatory effect of A-Exo. on axon growth

a, Representative (from > 3 replicates) immunoblot of APOE and APOJ in all eluted fractions (500 μl/fraction, pooled as indicated) of ACM (100 mL) from SEC with oversaturated exposure. 15 μl unconcentrated elution was run on immunoblot. **b**, Representative (from 3 replicates) ApoE immunoblot from *in vivo* isolated exosome fraction and flow-through samples following the SEC purification. P10 mice (1 mouse/sample) CNS tissues (brain and spinal cord) were used in exosome isolation. **c**, Quantification of βIII-tubulin^{+} neuronal axon length of cortical neurons following treatment with spinal cord (SC) A-Exo. or SC A-Exo. and hAPOE3 (20μg/mL). Number of neurons quantified in each group shown in the graph (6-8 neurons/replicate, 3 biological replicates)/group; **d**, Quantification of βIII-tubulin^{+} neuronal axon length following co-treatment of A-Exo. with hAPOB, hAPOJ, or hAPOE3, respectively. 1μg A-Exo. was used in treatment. hAPOB, hAPOJ, or hAPOE3 each was at 10 μg/mL dose. Number of neurons quantified in each group shown in the graph (7-9 neurons/replicate, 3 biological replicates)/group; **e**, Quantification of βIII-tubulin^{+} neuronal axon length following treatment of hHDL (10 μg/mL), hApoE3 (20 μg/mL), and cholesterol (1 μg/mL), respectively. Number of neurons quantified in each group shown in the graph (5-8 neurons/replicate, 2 biological replicates)/group; Representative images (**f**) and quantification (**g**) of βIII-tubulin^{+} neuronal axon (white arrows) length following co-treatment of A-Exo. and ApoE competitive receptor associated protein (RAP, 50 μg/mL). Scale bar: 100 μm; Number of neurons quantified in each group shown in the graph (5-8 neurons/replicate, 2 biological replicates)/group; **h**, Representative (from 3 replicates) ApoE immunoblot from WT or ApoE ACM (50μg proteins), and ApoE A-Exo (2 μg proteins).

i, Detection (from 5 replicates) of HepaCAM but not Apoe following HepaCAM immunoprecipitation from astrocyte lysates (50 μg proteins). 1μg A-Exo. was used in **c, d**, **f,** and **g**. p values in **c, d, e,** and **g** determined from one-way ANOVA followed by a Tukey post-hoc test. Data are presented as mean values +/- SEM.

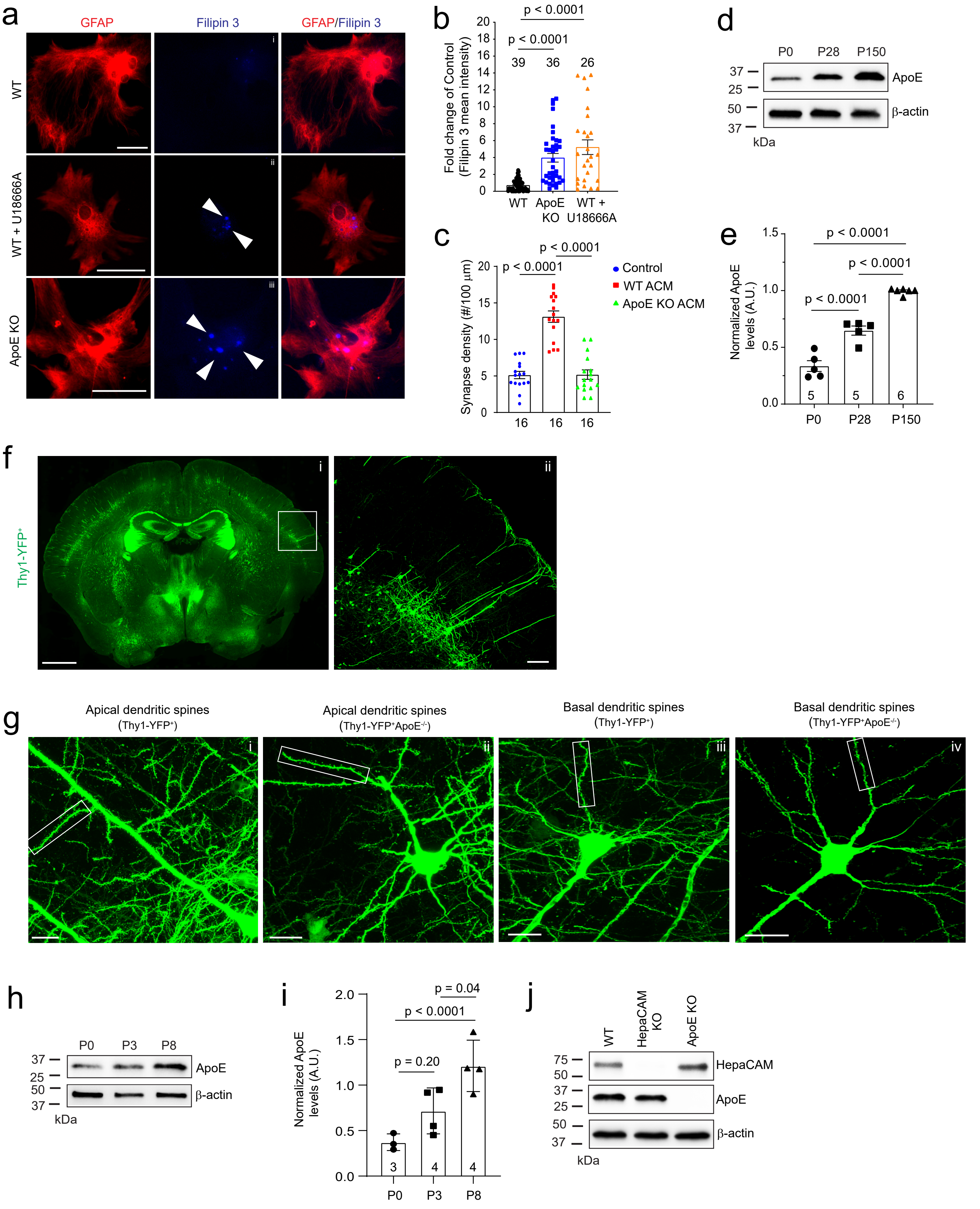

Supplementary Figure 7

Supplementary Fig. 7 ApoE deficiency reduces developmental dendritic spine formation on layer V cortical pyramidal neurons

Representative images (**a**) of cultured astrocytes and cholesterol labeling and quantification (**b**) of cholesterol in astrocytes based on Filipin 3 fluorescent intensity. Scale bar: 50 μm; White arrows: Filipin 3^+ cholesterol labeling; Number of astrocytes quantified in each group shown in the graph (8-12 neurons/replicate, 3 biological replicates)/group; **c**, Quantification of synapse density in neurons treated with WT and ApoE-deficient ACM. n = 16 neurons (8 neurons/replicate, 2 biological replicates)/group; Representative images (**d**) of ApoE immunoblot and quantification (**e**) of ApoE expression in the cortex during postnatal development; n = 5 mice for P0 and P28, 6 mice for P150; **f**, YFP labeling of neurons and neurites in Thy1-YFP^+ mice. Subpanel i: Representative (from > 5 mice) image of coronal section of the Thy1-YFP^+ mouse brain (scale bar: 1mm); ii: a magnified view of the motor cortex (white box) in the subpanel i (scale bar: 100 μm); **g**, Representative (from > 20 neurons) images of YFP^+ neurons and their dendritic spines. Subpanel i: apical dendritic spines from Thy1-YFP^+ mice; ii: apical dendritic spines from Thy1-$YFP^+ApoE^{-/-}$ mice; iii: basal dendritic spines from Thy1-YFP^+ mice; iv: basal dendritic spines from Thy1-$YFP^+ApoE^{-/-}$ mice; Scale bars: 20 μm; a magnified view of the highlighted box is shown in Fig. 7d-e; Representative ApoE immunoblot (**h**) and quantification (**i**) from spinal cords at P0, P3, and P8. n = 3 mice for P0, 4 mice for P3 and P8; **j**, Representative (from 3 replicates) HepaCAM and ApoE immunoblots from cortex of ApoE KO and HepaCAM KO mice at P30. p values in **b, c, e,** and **i** determined from one-way ANOVA followed by a Tukey post-hoc test. Data are presented as mean values +/- SEM.

Supplementary Table 1
Detected proteins with at least 3 peptides and mean iBAQ values > 1 x 10^5

Gene symbol	Entrez gene name	Location	Type(s)	Mean iBAQ value
CNTN1	contactin 1	Plasma Membrane	enzyme	756000
GNAI2	G protein subunit alpha i2	Plasma Membrane	enzyme	26100000
GNAO1	G protein subunit alpha o1	Plasma Membrane	enzyme	16800000
GNB1	G protein subunit beta 1	Plasma Membrane	enzyme	15400000
GNB2	G protein subunit beta 2	Plasma Membrane	enzyme	18000000
PLSCR3	phospholipid scramblase 3	Plasma Membrane	enzyme	529000
EDNRB	endothelin receptor type B	Plasma Membrane	G-protein coupled receptor	2770000
GPRC5B	G protein-coupled receptor class C group 5 member B	Plasma Membrane	G-protein coupled receptor	36600000
GPM6A	glycoprotein M6A	Plasma Membrane	ion channel	141000000
TTYH1	tweety family member 1	Plasma Membrane	ion channel	19600000
TTYH3	tweety family member 3	Plasma Membrane	ion channel	1030000
DDR1	discoidin domain receptor tyrosine kinase 1	Plasma Membrane	kinase	647000
APP	amyloid beta precursor protein	Plasma Membrane	other	1490000
CD109	CD109 molecule	Plasma Membrane	other	327000
CD63	CD63 molecule	Plasma Membrane	other	19000000
CD81	CD81 molecule	Plasma Membrane	other	186000000
CD9	CD9 molecule	Plasma Membrane	other	195000000
CSPG4	chondroitin sulfate proteoglycan 4	Plasma Membrane	other	2790000
HEG1	heart development protein with EGF like domains 1	Plasma Membrane	other	22600000
HEPACAM	hepatic and glial cell adhesion molecule	Plasma Membrane	other	3280000
IGSF8	immunoglobulin superfamily member 8	Plasma Membrane	other	14800000
ITM2B	integral membrane protein 2B	Plasma Membrane	other	1480000
ITGA3	integrin subunit alpha 3	Plasma Membrane	other	5510000
ITGB5	integrin subunit beta 5	Plasma Membrane	other	534000
IFITM3	interferon induced transmembrane protein 3	Plasma Membrane	other	76700000
NCAM1	neural cell adhesion molecule 1	Plasma Membrane	other	1340000
PROM1	prominin 1	Plasma Membrane	other	15300000
TNR	tenascin R	Plasma Membrane	other	121000000
TSPAN14	tetraspanin 14	Plasma Membrane	other	3530000
TSPAN6	tetraspanin 6	Plasma Membrane	other	20900000
TSPAN7	tetraspanin 7	Plasma Membrane	other	51500000
TSPAN9	tetraspanin 9	Plasma Membrane	other	5950000
THY1	Thy-1 cell surface antigen	Plasma Membrane	other	10900000
ADAM10	ADAM metallopeptidase domain 10	Plasma Membrane	peptidase	5450000
PTPRZ1	protein tyrosine phosphatase receptor type Z1	Plasma Membrane	phosphatase	235000000
NOTCH1	notch receptor 1	Plasma Membrane	transcription regulator	279000
CD14	CD14 molecule	Plasma Membrane	transmembrane receptor	171000
IGF2R	insulin like growth factor 2 receptor	Plasma Membrane	transmembrane receptor	161000
ITGA6	integrin subunit alpha 6	Plasma Membrane	transmembrane receptor	586000
ITGAM	integrin subunit alpha M	Plasma Membrane	transmembrane receptor	2080000
ITGAV	integrin subunit alpha V	Plasma Membrane	transmembrane receptor	745000
ITGB1	integrin subunit beta 1	Plasma Membrane	transmembrane receptor	11100000
ITGB2	integrin subunit beta 2	Plasma Membrane	transmembrane receptor	2600000
LRP1	LDL receptor related protein 1	Plasma Membrane	transmembrane receptor	5960000
PLXNB1	plexin B1	Plasma Membrane	transmembrane receptor	122000
PLXNB2	plexin B2	Plasma Membrane	transmembrane receptor	1330000
SSC5D	scavenger receptor cysteine rich family member with 5 domains	Plasma Membrane	transmembrane receptor	1260000
ABCA1	ATP binding cassette subfamily A member 1	Plasma Membrane	transporter	865000
ATP1A1	ATPase Na+/K+ transporting subunit alpha 1	Plasma Membrane	transporter	24100000
ATP1B1	ATPase Na+/K+ transporting subunit beta 1	Plasma Membrane	transporter	6720000
ATP1B2	ATPase Na+/K+ transporting subunit beta 2	Plasma Membrane	transporter	2000000
ATP1B3	ATPase Na+/K+ transporting subunit beta 3	Plasma Membrane	transporter	3380000
ATP2B4	ATPase plasma membrane Ca2+ transporting 4	Plasma Membrane	transporter	351000
BSG	basigin (Ok blood group)	Plasma Membrane	transporter	7200000
GJA1	gap junction protein alpha 1	Plasma Membrane	transporter	880000
LDLR	low density lipoprotein receptor	Plasma Membrane	transporter	6350000
MLC1	modulator of VRAC current 1	Plasma Membrane	transporter	3230000
SLC9A3R1	SLC9A3 regulator 1	Plasma Membrane	transporter	6680000
SLC1A3	solute carrier family 1 member 3	Plasma Membrane	transporter	17900000
SLC2A1	solute carrier family 2 member 1	Plasma Membrane	transporter	5160000
SLC3A2	solute carrier family 3 member 2	Plasma Membrane	transporter	6040000
SLC38A3	solute carrier family 38 member 3	Plasma Membrane	transporter	7400000
SLC44A1	solute carrier family 44 member 1	Plasma Membrane	transporter	6210000
SLC7A2	solute carrier family 7 member 2	Plasma Membrane	transporter	474000
Slco1a4	solute carrier organic anion transporter family, member 1a4	Plasma Membrane	transporter	2360000
STAB1	stabilin 1	Plasma Membrane	transporter	786000

Supplementary Table 1. Transmembrane proteins identified from A-Exo. by LC/MS/MS. Each identified protein has at least 3 peptide hits with 95% confidence threshold; The mean iBAQ value is greater than 1 x 10^5.
