# Supplementary material for: Astroglial exosome HepaCAM signaling and ApoE antagonization coordinates early postnatal cortical pyramidal neuronal axon growth and dendritic spine formation
Source: Nat Commun. 2023 Aug 24;14:5150. doi: 10.1038/s41467-023-40926-2 (PMC10449881; doi:10.1038/s41467-023-40926-2)
Supplement: Supplementary file 3 — Description of Additional Supplementary Files [file 41467_2023_40926_MOESM3_ESM.docx]

**Description of Additional Supplementary Files:**

**Supplementary Movie 1:** Live imaging of axon growth in control (untreated) primary cortical neuronal cultures

**Supplementary Movie 2:** Live imaging of axon growth in A-Exo. (1μg)-treated primary cortical neuronal cultures.
