# Supplementary material for: Astroglial exosome HepaCAM signaling and ApoE antagonization coordinates early postnatal cortical pyramidal neuronal axon growth and dendritic spine formation
Source: Nat Commun. 2023 Aug 24;14:5150. doi: 10.1038/s41467-023-40926-2 (PMC10449881; doi:10.1038/s41467-023-40926-2)
Supplement: Supplementary file 6 — Reporting Summary [file 41467_2023_40926_MOESM6_ESM.pdf]

nature portfolio

Corresponding author(s): Yongjie Yang

Last updated by author(s): Jul 23, 2023

Reporting Summary

Nature Portfolio wishes to improve the reproducibility of the work that we publish. This form provides structure for consistency and transparency in reporting. For further information on Nature Portfolio policies, see our Editorial Policies and the Editorial Policy Checklist.

Statistics

For all statistical analyses, confirm that the following items are present in the figure legend, table legend, main text, or Methods section.

n/a	Confirmed	
☐	☒	The exact sample size (*n*) for each experimental group/condition, given as a discrete number and unit of measurement
☐	☒	A statement on whether measurements were taken from distinct samples or whether the same sample was measured repeatedly
☐	☒	The statistical test(s) used AND whether they are one- or two-sided<br>*Only common tests should be described solely by name; describe more complex techniques in the Methods section.*
☒	☐	A description of all covariates tested
☐	☒	A description of any assumptions or corrections, such as tests of normality and adjustment for multiple comparisons
☐	☒	A full description of the statistical parameters including central tendency (e.g. means) or other basic estimates (e.g. regression coefficient) AND variation (e.g. standard deviation) or associated estimates of uncertainty (e.g. confidence intervals)
☐	☒	For null hypothesis testing, the test statistic (e.g. *F*, *t*, *r*) with confidence intervals, effect sizes, degrees of freedom and *P* value noted<br>*Give P values as exact values whenever suitable.*
☒	☐	For Bayesian analysis, information on the choice of priors and Markov chain Monte Carlo settings
☒	☐	For hierarchical and complex designs, identification of the appropriate level for tests and full reporting of outcomes
☒	☐	Estimates of effect sizes (e.g. Cohen's *d*, Pearson's *r*), indicating how they were calculated

Our web collection on statistics for biologists contains articles on many of the points above.

Software and code

Policy information about availability of computer code

Data collection	Izon qNano software (v 3.2), Nikon NIS-Elements (v 5.41), Leica LAS X (v 3.5.5), ZEN2 software (v 3.5), ChemiDoc imaging software (v 2.3), Proteome Discoverer software (v 2.4), StepOne Software (v 2.3)
Data analysis	Fiji Image J (v 2.9), Prism (v8 & v9), Scaffold software (v 5.1), ZEN2 software (v 3.5), Leica LAS X (v 3.5.5), StepOne Software (v 2.3), Ingenuity Pathway Analysis software (v 01-22-01), SynPAnal (v 1), Imaris imaging analysis software (v 9.7), G*power (3.1)

For manuscripts utilizing custom algorithms or software that are central to the research but not yet described in published literature, software must be made available to editors and reviewers. We strongly encourage code deposition in a community repository (e.g. GitHub). See the Nature Portfolio guidelines for submitting code & software for further information.

Data

Policy information about availability of data

All manuscripts must include a data availability statement. This statement should provide the following information, where applicable:

- Accession codes, unique identifiers, or web links for publicly available datasets
- A description of any restrictions on data availability
- For clinical datasets or third party data, please ensure that the statement adheres to our policy

All data supporting the findings of this study are available from the corresponding author on reasonable request. This is to protect data from free commercial use. In addition, as organizing and labeling the original data can be time consuming, it is best effective and useful for us to provide specific data upon request. The proteomics data of astroglial exosomes have

been deposited into ProteomeXchange (dataset identifier PXD040650; Project DOI: 10.6019/PXD040650). We also used the open database Swiss-Prot Mouse protein database to identify exosome proteomics.

Research involving human participants, their data, or biological material

Policy information about studies with human participants or human data. See also policy information about sex, gender (identity/presentation), and sexual orientation and race, ethnicity and racism.

Reporting on sex and gender	*Use the terms sex (biological attribute) and gender (shaped by social and cultural circumstances) carefully in order to avoid confusing both terms. Indicate if findings apply to only one sex or gender; describe whether sex and gender were considered in study design; whether sex and/or gender was determined based on self-reporting or assigned and methods used. Provide in the source data disaggregated sex and gender data, where this information has been collected, and if consent has been obtained for sharing of individual-level data; provide overall numbers in this Reporting Summary. Please state if this information has not been collected. Report sex- and gender-based analyses where performed, justify reasons for lack of sex- and gender-based analysis.*
Reporting on race, ethnicity, or other socially relevant groupings	*Please specify the socially constructed or socially relevant categorization variable(s) used in your manuscript and explain why they were used. Please note that such variables should not be used as proxies for other socially constructed/relevant variables (for example, race or ethnicity should not be used as a proxy for socioeconomic status). Provide clear definitions of the relevant terms used, how they were provided (by the participants/respondents, the researchers, or third parties), and the method(s) used to classify people into the different categories (e.g. self-report, census or administrative data, social media data, etc.) Please provide details about how you controlled for confounding variables in your analyses.*
Population characteristics	*Describe the covariate-relevant population characteristics of the human research participants (e.g. age, genotypic information, past and current diagnosis and treatment categories). If you filled out the behavioural & social sciences study design questions and have nothing to add here, write "See above."*
Recruitment	*Describe how participants were recruited. Outline any potential self-selection bias or other biases that may be present and how these are likely to impact results.*
Ethics oversight	*Identify the organization(s) that approved the study protocol.*

Note that full information on the approval of the study protocol must also be provided in the manuscript.

Field-specific reporting

Please select the one below that is the best fit for your research. If you are not sure, read the appropriate sections before making your selection.

☒ Life sciences ☐ Behavioural & social sciences ☐ Ecological, evolutionary & environmental sciences

For a reference copy of the document with all sections, see nature.com/documents/nr-reporting-summary-flat.pdf

Life sciences study design

All studies must disclose on these points even when the disclosure is negative.

Sample size	Sample size and biological replicates for each experiment is described in figure legends. Previous results and the G*Power (version 3.1) were used to estimate the reasonable sample size. data from both sexes of mice were collected and analyzed together, as there is no evident differences observed between male and female mice.
Data exclusions	The robust regression and outlier removal (ROUT, 1%) method in Prism has been used to exclude outliers.
Replication	We have been using reagents that have been verified. We have repeated all experimental findings sufficiently, as described in the experimental design and figure legend. In addition, we have used both mouse and human models with different genotypes to validate our results.
Randomization	All experimental groups are always randomly assigned.
Blinding	All experiemnts were carried out as blind as possible with all controls included. Data analysis was done blindly by different lab members.

Reporting for specific materials, systems and methods

We require information from authors about some types of materials, experimental systems and methods used in many studies. Here, indicate whether each material, system or method listed is relevant to your study. If you are not sure if a list item applies to your research, read the appropriate section before selecting a response.

Materials & experimental systems

n/a	Involved in the study
	☒ Antibodies
☒	☐ Eukaryotic cell lines
☒	☐ Palaeontology and archaeology
	☒ Animals and other organisms
☒	☐ Clinical data
☒	☐ Dual use research of concern
☒	☐ Plants

Methods

n/a	Involved in the study
☒	☐ ChIP-seq
☒	☐ Flow cytometry
☒	☐ MRI-based neuroimaging

Antibodies

Antibodies used

All antibodies used in the study has been clearly described in the materials and methods, including the catalog number, vendor, and use dilution.
For immunoblotting, the following primary antibodies were used: Thrombospondin (TSP)-1 (1:100, Santa Cruz clone SC-8), Thrombospondin (TSP)-2 (1:100, BD Biosciences), Hevin (1:200, R&D Systems), Sparc (1:200, R&D Systems), Sema3a (1:200, clone A-18 Santa Cruz), TSG101 (1:100, clone C-2 Santa Cruz), GFAP (1:2000, Dako, #Z0334), TurboGFP (1:2000, Evrogen, #AB513), mouse CD63 (1:200, MBL, # D263-3), CD81(1:1000, clone B-11, Santa Cruz), Alix (1:1000, Santa Cruz, clone 1A12, #sc-53540), GM130 (1:1000, Abclonal, #A5344), Calregulin (1:100, Santa Cruz, clone F-4 #sc-373863), HistoneH2A (1:400, Cell Signaling, #2718), GLAST (1:1000, GeneTex, #GTX134060), β-actin (1:1000, A1978, Sigma), HepaCAM N-terminal antibody (1:500, ProteinTech), HepaCAM C-terminal antibody (1:200, Affinity Biosciences, #DF12075), ApoE (1:1000, ABclonal, #A16344), ApoB (1:500, ABclonal, #A4184), ApoJ (1:500, ABclonal, #A1472). Secondary antibodies, including ECL anti-mouse IgG (1:10000, GE HealthCare NA931V), anti-rabbit IgG-HRP (1:5000, GE Health Care NA934V), mouse anti-Goat IgG-HRP (1:1000, Santa Cruz) and anti-Rat IgG-HRP (1:5000, Thermo Fisher Scientific SC-2357);
For immunoprecipitation, Dynabeads® M-270 Epoxy beads (Thermo Fisher Scientific) with anti-CD81 (clone Eat-2, BioLegend), anti-HepaCAM (Affinity Biosciences, # DF12075), and mouse IgG1 (clone MG1-45 BioLegend) were used.
For immunostaining, The following antibodies were used: β-III tubulin (1:1000, MAB1195, R&D system), rabbit anti-MAP2 (1:1000, GeneTex, GTX133109), Gap43 Antibody (1:500, Novus Biologicals, clone 2G13), anti-mouse Tau (1:500, GeneTex), mouse anti-Map2 (1:1000, Sigma, M9942), rat anti-GFAP (1:5000, zymed, 273756), rabbit anti-GFAP (1:1000, Dako), and anti-human Tau (1:500, Dako), GFAP (1:5000, Dako, #Z0334), mCD63 (1:50, MBL, #D263-3), and HepaCAM (1:200, R&D, #MAB4108). Primary antibodies were visualized with appropriate secondary antibodies conjugated with Alexa fluorophores (1:1000 Invitrogen).

Validation

All antibodies especially primary antibodies have been previously validated based on the manufacture's statement on the website or validated in the literature, or in our study with the use of knock-out mice.

Animals and other research organisms

Policy information about studies involving animals; ARRIVE guidelines recommended for reporting animal research, and Sex and Gender in Research

Laboratory animals

hCD63-GFP floxed mice (C57BL6) were generated in the lab by homologous recombination. The WT (C57B/6J, #000664), Ai14-tdTf/f reporter (#007914), Tg(Slc1a3-cre/ERT)1Nat/J (#012586), ApoE-KO (B6.129P2-Apoe tm1Unc/J #002052), B6.Cg-Tg (Thy1-YFP)HJrs/J (#003782), and B6.C-Tg(CMV-Cre)1Cgn/J(#006054) mice were obtained from the Jackson Laboratory. HepaCAM knock-out (KO) mice were generated by breeding HepaCAM floxed mice (a kind gift from Dr. Cagla Eroglu at Duke University) with CMV-Cre mice. Both male and female mice were used in all experiments. Both sexes were used in all studies. All mice were maintained on a 12 h light/dark cycle with food and water ad libitum.

Wild animals

no wild animals were used in the study.

Reporting on sex

Both male and female mice were used in all experiments. No sex-specific differences were observed, as a result, data from both sexes were grouped together for reporting.

Field-collected samples

no field collected samples were used in the study.

Ethics oversight

Tufts University IACUC.

Note that full information on the approval of the study protocol must also be provided in the manuscript.
